# The ageing virus hypothesis: Epigenetic ageing beyond the Tree of Life

**DOI:** 10.1002/bies.202400099

**Published:** 2024-10-14

**Authors:** Éric Bapteste

**Affiliations:** ^1^ Institut de Systématique, Évolution, Biodiversité (ISYEB), Sorbonne Université CNRS, Museum National d'Histoire Naturelle, EPHE Université Des Antilles Paris France

**Keywords:** age‐distorter, ageing, epigenetics, evolution, viruses

## Abstract

A recent thought‐provoking theory argues that complex organisms using epigenetic information for their normal development and functioning must irreversibly age as a result of epigenetic signal loss. Importantly, the scope of this theory could be considerably expanded, with scientific benefits, by analyzing epigenetic ageing beyond the borders of the Tree of Life. Viruses that use epigenetic signals for their normal functioning may also age, that is, present an increasing risk of failing to complete their individual life cycle and to disappear with time. As viruses are ancient, abundant, and infect a considerable diversity of hosts, the ageing virus hypothesis, if verified, would have important consequences for many fields of the Life sciences. Uncovering ageing viruses would integrate the most abundant and biologically central entities on Earth into theories of ageing, enhance virology, gerontology, evolutionary biology, molecular ecology, genomics, and possibly medicine through the development of new therapies manipulating viral ageing.

## Graphical abstract

Viruses would age when the epigenetic signals they need to carry out their life cycle are degraded. Given the abundance and age of viruses, epigenetic aging of viruses would extend theories on the evolution of aging beyond cellular organisms, and encourage new models of viral dynamics and new anti‐viral therapies.

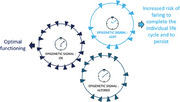

## 1 INTRODUCTION

Understanding how, when, and why organisms age is a major scientific issue, with considerable medical and social implications. Consequently, scientists have produced multiple theories to unveil the mechanistic causes as well as the evolutionary causes of ageing.^[^ ^1^, ^2^, ^3^, ^4^, ^5^, ^6^, ^7^ ^]^ Mainstream theories are often complementary, but they share common features^[^ ^1^ ^]^ that determine their application scope. In particular, mainstream theories of the evolution of ageing^[^ ^3^, ^4^, ^5^ ^]^ were clearly developed to explain the ageing of cellular lineages, most commonly metazoans and mammals. Accordingly, most experimental studies of ageing are conducted on a few eukaryotic model species sampled within Drosophila, Nematodes, and yeasts.^[^ ^7^, ^8^, ^9^, ^10^, ^11^ ^]^ However, *contra* historical ideas of Weissmann,^[^ ^12^ ^]^ many experts now consider that ageing is not restricted to Eukaryotes.^[^ ^13^, ^14^, ^15^, ^16^, ^17^ ^]^ Some prokaryotic species age, although this phenotype has so far only been experimentally characterized for a few bacterial species.^[^ ^17^, ^18^, ^19^, ^20^ ^]^ The idea that ageing is a widespread, although not universal,^[^ ^21^ ^]^ phenomenon across the Tree of Life is therefore well‐admitted, but many causes of ageing within the Tree of Life likely remain to be characterized.

Excitingly, in the past decade, the idea that ageing could be mechanistically connected to epigenetic signal dynamics has gained traction,^[^ ^22^, ^23^, ^24^, ^25^, ^26^ ^]^ although one may argue that organisms with negligible senescence, such as turtles or naked mole rats^[^ ^27^, ^28^ ^]^ may perhaps be immune to this mode of ageing. Indeed, the ageing process results in significant epigenetic changes at all levels of chromatin and DNA organization. These include reduced global heterochromatin, nucleosome remodeling, and loss, changes in histone marks, global DNA hypomethylation with CpG island hypermethylation, and the relocalisation of chromatin modifying factors.^[^ ^29^ ^]^ Accordingly, a thought‐provoking theory of ageing, hereafter called the “epigenetic theory of ageing” (ETA) was recently proposed. ETA mainly intends to explain ageing in mammals and possibly in other “complex” organisms, that is, multicellular eukaryotes, that use epigenetic information for their normal development and/or functioning.^[^ ^30^ ^]^ ETA states that in order to avoid ageing as a result of epigenetic signal loss, any system that relies on epigenetics must guarantee sufficient epigenetic fidelity during its life cycle. Indeed, unless epigenetic damage, for example, the gain or loss of a methyl group along the DNA molecule, can be faithfully fixed, it will result in information loss. However, there would be only two theoretical ways to fix epigenetic damage with accuracy: either a back‐up exists that can be copied to restore the proper epigenetic pattern, or a repair algorithm can be activated to produce the same result.^[^ ^30^ ^]^

Duffield et al. argue that cells from complex organisms in particular (according to them multicellular eukaryotes) are unfortunately unlikely to harbour these kinds of solutions.^[^ ^30^ ^]^ Consider a hemi‐methylated DNA region (as opposed to a fully methylated or non‐methylated DNA) within such a cell that resulted from an erroneous incorporation or loss of a methyl group on one of the two DNA strands. The focal cell with such a hemi‐methylated DNA region does not contain a second epigenetically marked DNA molecule that it could use as a master copy to determine how this hemi‐methylated pattern should be resolved. Moreover, it is also unlikely that the correct information on how to fix this epigenetic pattern can be found encoded on the cell DNA, as records of this specific information would require to encode which DNA sites/regions must be methylated or unmethylated at any stage of the cell cycle, an amount of information presumably overwhelming the coding capacities of the DNA. Instead, Duffield et al. propose that a repair system of epigenetic signal might be encoded using a biological system with more immediately accessible memory and a possibly broader storage capacity than DNA, that is, encoded as epigenetic signal. However, this model entails that epigenetic information is threatened by lossy compression, inevitably making epigenetic repair systems sensitive to epigenetic signal loss, which eventually leads to a vicious circle of epigenetic failure and to cellular and organismal ageing.

While consistent with the mainstream theoretical framework on ageing and its evolution, ETA nonetheless introduces very original concepts with unparalleled potential to rethink ageing amongst epigenetically endowed evolving entities. Of note, ETA echoes, and was possibly inspired by “the (epigenetic) information theory of ageing” by Yang et al.^[^ ^31^ ^]^ These two theories agree that epigenetic changes with time, and more specifically that the loss of epigenetic information causes ageing and is a major driver of ageing in mammals. In details, these two theories are non‐exclusive, although their focus differ on what processes (a fundamental design flaw vs. (stochastic?) damages) cause such problematic epigenetic loss with time. Furthermore, both theories seem to make opposite predictions on whether some existing biological memory/process/back‐up could rescue such a detrimental epigenetic loss. The (epigenetic) information theory of ageing considers that some critical epigenetic information are likely recorded in DNA, albeit with a limited resolution, as suggested by the ability of neural nets to predict methylation patterns from DNA, by the ability of partial reprogramming to restore younger epigenetic state or by the similarity between tissue‐specific DNA methylation patterns between individuals, which suggests that the information needed to generate these methylation patterns was found in their single‐cell embryos. In other words, according to the (epigenetic) information theory of ageing some back‐up for certain aspects of an epigenetic profile with limited resolution may be present in some mammalian cells at least, whereas for ETA epigenetic signal loss cannot be fixed in mammals. A minimal consensus version of both theories would therefore assume that epigenetic changes cause ageing, unless some mechanisms allowing to dampen the fragility of epigenetic repair systems or some back‐ups may have evolved in a lineage. Even without necessarily embracing all the conclusions of ETA, for example, regarding whether no cellular back‐up of the proper epigenetic signal exists (as other authors have imagined that covalent modifications of DNA, protein binding to DNA, RNA‐guided chromatin modification factors, or established RNA‐DNA hybrids^[^ ^32^ ^]^ may play such a role), a take‐home message of ETA is that whenever critical functions are epigenetically encoded, these functions are at risk of getting lost, and fixing detrimental epigenetic losses requires dedicated robust repair systems.

If that is correct, it is important to realize that multicellular organisms are not the only biological entities that significantly rely upon epigenetic information for their optimal functioning. Typically, many microbes: bacteria, archaea, protists (unicellular eukaryotes) and, beyond these cellular life forms united in the Tree of Life, viruses, use epigenetic information for their normal functioning. Thus, for instance, DNA methylases have been reported in 93% of the bacterial and archaeal genomes,^[^ ^33^, ^34^ ^]^ and their functions have been related to vital, adaptive biological processes, such as conditioning DNA repair, restriction‐modification defence mechanisms, phase variation, and so on.^[^ ^33^ ^]^ Moreover, even though they are not comprised of multiple tissues as animals are, microbes unquestionably present a high complexity in their own ways, at the level of their inner molecular processes and sometimes through their collective interactions. Consequently, microbes that use epigenetics too may suffer from epigenetic signal loss and age. Therefore, I propose that it would be timely and scientifically fruitful to substantially expand the intended explanatory scope of the recent epigenetic theory of ageing notably within but even beyond the Tree of Life, in particular to apply it to viruses.

### 1.1 Defining viral ageing

Several cases of viral ageing deserve to be distinguished based on the level, that is, individual, for example, one viral particle or one viral genome, or populational, for example, all individual viruses emerging from a given clone within an infected cell or within an infected host or within an environmental niche, that is considered. More precisely, an individual virus is said to age when it presents an increasing risk of failing to complete its individual life cycle, for example, when it presents an increasing risk to become defective and/or to disappear with time. Such a form of ageing is a form of biological ageing, because that individual virus increasingly suffers from an irreversible loss of functions. One particular form of biological ageing may notably affect individual viruses. Namely, when an individual virus, for example, a provirus inserted in a host genome, increasingly fails to replicate, this virus suffers from a form of replicative ageing. The idea that individual viral particles can age is an important notion to put forward, and it seems to be supported by observations, such as the report of viral particles that have lost their replicative potential yet are able to survive in an environment (be it in nature or within an infected host) or in a genome, as illustrated by the discovery of defective viruses. Defective viruses, unable to complete a full replication cycle in the absence of a co‐infecting non defective helper,^[^ ^35^ ^]^ have indeed been described across the entire Tree of Life, from prokaryotes, in which many defective prophages are found in bacterial genomes in a state of mutational decay^[^ ^36^ ^]^ to humans, where abundant low frequency hyper‐edited viral genomes presumed to reflect defective viruses have been detected.^[^ ^37^ ^]^ A viral particle that would be replicatively ageing would in addition keep on ageing chronologically until it gets destroyed, for example, until this particle disappears or its viral sequence decays to the point it is virtually no longer transcribed.

At the population level, closely related viruses are said to age demographically when the proportion of aged (hence defective) individual viruses that composes a population of viruses born during the same infection increases to the point that their host cell or their local niche stops being productive of new virions and/or that the risk that this population gets extinct increases independently of external changes in the selective pressures (e.g., in absence of changes in the immune response exerted by its host or of changes in its environmental niche). This definition, directly inspired from works by demographers, relies upon an inclusive definition of demographic ageing that considers ageing as change in the risk of dying over age within a given time period from conception to death, and assumes that individual viruses are born as a result of a replication event and that they die when their genetic information has so decayed that the virus can no longer be transcribed. Accordingly, a clonal population of viruses could be said to age reproductively when its productivity of new virions decreases with time independently of changes in the selective pressures exerted by its host or by its environmental niche.

Importantly, the above descriptions of individual ageing do not involve that any deleterious change, genetic or epigenetic, should count as ageing, which would dissolve the originality of the concept presented next. Indeed, what makes the concept of epigenetic ageing of viruses that will be developed below original is the nature of the mechanisms that ultimately trigger the deleterious changes in question. In the case of epigenetic ageing, the trigger is the loss of epigenetic signal. Accordingly, if these diverse instances of individual biological ageing, of individual replicative ageing and of demographic ageing at the populational level have their ultimate cause in an irreversible loss of viral epigenetic signal affecting individual viruses, then all these instances of viral ageing can in addition be considered as manifestations of epigenetic ageing. One might assume that viral populations may simply escape epigenetic signal loss since epigenetics resetting would occur in every cycle of virus propagation. However, if this were not the case, and viral particles failed to reset their epigenetic signals, or failed to produce viral particles with properly reset epigenetic signals, individual viral particles would have aged epigenetically, which may affect their populations. Consistently, epigenetically ageing individual viruses and epigenetically ageing populations of viruses would manifest altered, disabling epigenetic profiles with respect to younger viruses and younger viral populations. Of note, just like cellular organisms could be affected by a diversity of ageing mechanisms, among which epigenetic signal loss is one, as illustrated by the famous hallmarks of ageing proposed for mammals,^[^ ^7^ ^]^ viruses may age too due to different mechanisms, which are not the focus of the present paper, uniquely devoted to epigenetic viral ageing.

### 1.2 Justification of the proposed theoretical extension

### 1.3 Why viruses?

Viruses are amongst the oldest evolving biological forms on Earth.^[^ ^38^ ^]^ Estimates of the number of viral particles, limited to prokaryotic viruses, that is, phages, further suggest that viruses are also the most abundant biological forms, outnumbering bacterial and archaeal cells by at least one order of magnitude.^[^ ^39^ ^]^ Viruses are also ubiquitous components of the Web of Life.^[^ ^40^ ^]^ Consistently, the critical ecological and evolutionary roles of viruses are increasingly recognized. Viruses affect their host community dynamics ‐from prokaryotes to animals‐ and possibly impact even biogeochemical cycles.^[^ ^41^, ^42^, ^43^ ^]^ However, despite this genuine ubiquity and centrality in the biological world, viruses are currently largely overlooked by theories of ageing and by evolutionary theories of ageing. Thus, the Disposable Soma theory explains ageing as a trade‐off between reproduction and repair based on a neat differentiation between germen and soma. Consequently, this theory simply does not consider viruses as potential ageing agents.^[^ ^5^ ^]^ Both the Mutation Accumulation theory^[^ ^3^ ^]^ and the Antagonistic Pleiotropy^[^ ^4^ ^]^ theory are grounded on the concept of selection shadow : the idea that the strength of natural selection acting on organisms decreases past their reproductive peak during adulthood.^[^ ^2^ ^]^ If one considers that viruses lifecycle ends or starts with events of viral replication, there seems to be little to no room for selection shadow to impact viruses’ fitness, and for viruses to age according to these theories. Because viruses are not considered as ageing entities in their own right, they do not feature as mainstream ageing entities in these classic evolutionary theories of ageing. However, viruses have recently been proposed to play a role in the ageing of their hosts as candidate age‐distorters.^[^ ^44^, ^45^ ^]^ This latter notion warrants an extension of the classic theories of ageing to include co‐evolution events, arms races and symbioses between viruses and hosts as potential additional causes of organismal ageing, but still the notion of age‐distorters does not consider that viruses too may age.

By contrast, diving into the model of the ETA proposed by Duffield et al. inspires one to expand its scope beyond “complex systems and organisms,” in particular to viruses. In a nutshell, according to ETA, organisms that rely on epigenetic programs for their growth, reproduction or survival would be doomed to age because epigenetic signals can only be irreversibly lost, and therefore cellular and by extension organismal optimal functioning cannot be maintained indefinitely. More precisely, epigenetic signal loss would be a particularly acute problem for multicellular organisms because their organizational complexity would impose that potential repair systems that may rescue epigenetic signal loss could not be fully encoded in DNA. Rather, hypothetical repair systems would have to be epigenetically encoded, a problematic design that would make these hypothetical epigenetic‐based repair systems sensitive to the very phenomenon, that is, epigenetic signal loss, that they are supposed to counteract.

Therefore, unless viruses that use epigenetic signals for their normal functioning have evolved ways to fix epigenetic signal loss, the above considerations make them at risk to fall prey to such a loss and to detrimental dysfunctions. Consistently, it has been long known^[^ ^46^ ^]^ that some viruses rely upon dynamic epigenetic patterns at the DNA and/or at the RNA levels for their normal functioning. For instance, giant viruses carry DNA methyltransferases that enable them to regulate their replication cycle, fulfilling functions beyond the traditionally accepted role of epigenetic marks in restriction‐modification systems in bacteria and viruses, that is, identification or protection against foreign DNA entering a cellular host. Methylation of giant virus genomes is widespread, observed in 2 out of 3 giant virus families, although non‐uniformly distributed within these families. Most of their methyltransferases are conserved, functional, under purifying selection. These features suggest that they play a role in the evolutionary success of these viruses, because of their involvement in various interactions between viruses and other pathogens co‐infecting the same host. Moreover, these methyltransferases can circulate between the DNA of giant viruses, bacteria and their eukaryotic hosts, confirming the importance of such genetic instructions in the struggle for life.^[^ ^47^ ^]^ In addition, some viruses, such as SV40 viral particles, can carry epigenetic information in their chromatin, and transmit it from one virus generation to the next.^[^ ^48^ ^]^ This information is essential for the successful replication of these viruses, which must present multiple epigenomes at the same time and in the same cell to complete their life cycle. Moreover, the human Papillomavirus changes its epigenetic information in a region of its genome called LCR. Initially methylated, it becomes unmethylated during the stage of cell differentiation and viral production, while a more specific subregion, the E2 protein attachment site, becomes hypermethylated. Similarly, methylation of specific genes is involved in the transition from the lytic to the latent phase for the Epstein Barr Virus (EBV), with hypermethylation of the viral Cp and Wp promoters repressing production of the majority of the virus nuclear antigens.^[^ ^49^ ^]^ Such methylation changes, potentially masking the presence of viruses to the host immune system, have a major functional impact on the biology of these viruses.^[^ ^50^ ^]^ Furthermore, for DNA viruses such as adenoviruses and polyomaviruses, *de novo* methylation begins when they become latent, usually first in limited regions and then throughout the viral DNA. These methylations are often site‐specific, regulating late viral gene expression in a way that is reversible, particularly at viral gene promoters.^[^ ^49^ ^]^ Finally, the HIV‐1 RNA virus, before being integrated into the DNA of its host, is loaded with the histone variant H3.3 carrying mainly an epigenetic mark of repression: H3K9me3.^[^ ^51^ ^]^ Then, in its DNA‐integrated form, DNA methylations of this virus lock the expression of its genes over the long term, stabilizing its latency in the host.^[^ ^52^ ^]^ This latency enabled by epigenetics offers an advantage to the viruses concerned: they can establish durable reservoirs in their hosts, making their eradication impossible.^[^ ^51^ ^]^

One may wonder when epigenetic ageing induced by epigenetic signal loss may affect viruses, and whether some stages of the viral life cycle, for example, latent versus lytic stages or the first versus the last viruses released in the environment, are more at risk to result in aged viruses. All stages of the viral cycle seem to have their own risk to be affected by some epigenetic ageing. This is because viral epigenetic states have been demonstrated to play a role throughout the entire life cycle of viruses:^[^ ^53^, ^54^ ^]^ some epigenetic information is critical at the latent stage (to hide from the host immune system or to establish viral residency) or at the lytic stage, depending on the virus, or even critical for the transition between these two stages.^[^ ^55^ ^]^ Latent viruses, especially those that stay for decades in their hosts, certainly rely on their epigenetics for periods that exceed the time of individual viral replication and/or of the first release of replicated viral particles into the environment. Consistently, epigenetic ageing of individual viruses has potential to generate defective aged viruses that may eventually accumulate over time in their host cells. For instance, the KSHV virus is subject to progressive epigenetic inactivation,^[^ ^55^ ^]^ a form of epigenetic drift that restricts this virus ability to reactivate lytic transcription, as very few genomes within each infected cells (only 6 to 10) gave rise to a viral progeny. Hence, I propose that, while most viruses are known to spontaneously generate defective viral genomes due to errors during replication, epigenetic signal loss may be another possible trigger of viruses defectivity.

Importantly, owing to the huge environmental viral diversity, in particular within the microbial dark matter,^[^ ^56^ ^]^ future analyses of viral epigenetics are likely to reveal more viruses that use epigenetic signals to sustain their replication and/or survival. In such viruses, epigenetic signal loss could therefore theoretically result in a form of replicative ageing and of biological ageing, respectively. Populations of free environmental virions that are losing their epigenome integrity, possibly due to spontaneous chemical reactions, may also have their infective potential affected by epigenetic loss.

If some epigenetic signal carried by viruses is likely to be lost with time, this should in particular occur when the DNA of viruses becomes intermingled with the DNA of their hosts, starting with mammalian viruses. Typically, during DNA replication, a process that generates two hemi‐methylated DNA copies, the presence of oxidized 5mC bases could typically promote the loss of 5mCs, as DNMT1 enzymes, capable of restoring the epigenetic signal during and after DNA replication, are less efficient on this type of base, and perhaps less spontaneously mobilized to repair the damaged viral epigenome than that of the host.^[^ ^57^ ^]^

In fact, the quality and stability of the viral epigenetic signal is likely to be especially constrained by the interactions between a virus and its host. Infections are critical periods during which a virus epigenetic profile has chances to be altered. This is especially obvious when viruses rely on the host machinery to establish and to maintain their epigenetic profiles or when the host actively shapes the epigenetic information of its viruses. Under that hypothesis, when an optimal epigenetic profile in a virus results from a tight co‐evolution between this virus and its hosts, the functionality of the host, notably determined by the biological age of the host, appears as a possible important parameter to generate viral epigenetic signal with high fidelity. A biologically aged host, with impaired molecular and cellular processes, may less effectively maintain the optimal epigenetic signal of its viruses and contribute to the ageing of its individual viral particles and of its viral populations when its own defective host machinery is hijacked by viruses. Simply put, when integrated viruses rely on the (imperfect) repair mechanisms of their hosts' epigenetic systems to maintain their own epigenetic condition in the face of chemical accidents, once these viruses have boarded the host DNA, their longevity partly depends on the proper functioning of the epigenetic repair systems of their hosts, which would be limited according to ETA.^[^ ^30^ ^]^

Third, even without embracing ETA, according to classic evolutionary theories of ageing, the older a mammalian host gets, the more natural selection turns a blind eye to its fate,^[^ ^1^, ^2^ ^]^ and the less efficiently any host repair system is likely to operate. Therefore, viruses lurking in an old host, and depending from the functioning of this host for their own preservation, could age due to selection shadow. This view is highly hypothetical and may seem counterintuitive, considering that some latent viruses (and transposons) often tend to reactivate with host age, but not always.^[^ ^58^ ^]^ However, such a reactivation may not be beneficial to the viruses, even in the long‐run, because it can culminate in the death of their hosts and play against the viruses survival. Viral reactivation in an aged host may thus reflect a failure to maintain an optimal long‐term survival strategy for a virus and, as such, may appear as a manifestation of viral ageing rather than as a manifestation of a preserved virus functionality. Indeed, the evolutionary benefits for the virus in case of viral reactivation are not clear, and some authors have suggested that reactivation in aged hosts may not be part of a selected strategy for the virus.^[^ ^59^ ^]^ In the case of oncogenic viruses, as latency limits the risk of pathology,^[^ ^60^ ^]^ latency rather than reactivation may benefit both the hosts and the virus. In the case of HSV‐1 infection, a virus that is not considered oncogenic but that may increase risk of malignant progression, latency also appears as an adaptive phenotype that allows the virus to escape immune host response.^[^ ^61^ ^]^ Reactivation is therefore possibly not advantageous for these viruses. Likewise, spontaneous lytic reactivation of the KSHV occurs at a low rate in latently infected cells in disease and culture, which suggests an imperfect epigenetic maintenance of viral transcription programs.^[^ ^55^ ^]^

Moreover, viral reactivation with host age may hide another important phenomenon suggestive of viral ageing: namely the dynamics of the proportion of defective viruses in infected cells/hosts with host age. If an aged host induces the ageing of its resident viruses as is hypothesized here, and if ageing viruses being less functional tend to become defective, then infected ageing host cells should contain more defective viruses with time. While non defective resident viruses could still reactivate because their host immune system is weaker, these reactivations may originate from viral populations increasingly loaded with defective viruses, hence involve aged populations of viruses.

### 1.4 Possible payoffs to uncover ageing viruses

If the « ageing virus hypothesis » holds, biological entities that uncontroversially evolve outside the Tree of Life and critically shape the Web of Life^[^ ^62^ ^]^ would also deserve to be considered as ageing. This would dramatically expand the phylogenetic scope of ageing studies. If individual viruses within a host or in the environment present an increasing risk to not complete their life cycles, for example, to become defective, to fail to replicate and/or to disappear with time, as a result of their loss of epigenetic information, this phenotype of viral ageing would deserve to be explicitly modelled to enhance explanations of viral dynamics both in nature and across infected host genomes. In particular, the question of “why would viruses age” could also be addressed at the level of their populations. In brief, three possible kinds of consequences of the ageing of viral particles on their populations would be possible. First, the potential consequences on a viral population of the individual loss of epigenetic information could be harmless, in particular if epigenetic changes are outpaced by the frequency of mutations and by the speed of evolution. Second, epigenetic ageing of individual viral particles may be a selected/collateral damage that supports the viral population. This would be the case if defective viral particles inevitably accompanied the rejuvenation of viral population and supported their further evolutionary potential. Interestingly, defective prophages have been documented to contribute important biological properties to their bacterial hosts.^[^ ^36^ ^]^ If some of these defective viruses were thus induced by epigenetic changes under the control of populational mechanisms, epigenetic ageing might be seen by some as a form of asymmetry within a viral population, because individual particles would age but viral populations would not. Third, epigenetic ageing of individual viral particles may be harmful for viral populations. When the proportion of aged viral particles within a viral population gets over a certain threshold, for example, within an infected cell or within an infected host, the viral population is at risk to collapse, or to threaten an eventual stability within its host (e.g., in the case of viral reactivation), leading to a decrease in the virus fitness either by causing the death of its own host or by triggering the action of the host immune defence against its infective viral populations, including against non‐aged viral particles.^[^ ^63^ ^]^ Empirical experiments would be needed to conclude, for specific viruses, what situation occurs.

Consistent with the notion that some viruses age, viruses integrated into the DNA of their hosts often fail to complete their viral cycle^[^ ^54^ ^]^ and their host genomes turn into their graves, perhaps because some viruses have become epigenetically old? If so, it is important to determine how many old viruses our genome contains. Likewise, it becomes important to learn how many sub‐performant, aged viruses, as opposed to viruses with full replicative potential, are circulating in the environment.

The co‐evolution of ageing viruses and ageing hosts would also deserve further clarifications. If viruses suffer from an epigenetic Achilles’ heel, and their functioning can be altered when they lose epigenetic information, evolution may have favored, in certain host lineages invaded by such viruses, hosts capable of actively ageing the particles and/or populations of their intrusive invaders. An evolutionary advantage for hosts with a molecular machinery capable of ageing their intruders by “rotting their epigenetics,” over and above putting a spell on their enemies, could be to capture viral genetic information that has become harmless, which these hosts could recycle to perform other functions. Alternatively, as ageing viruses would by definition lose some of their functions, in the case of ageing mutualistic viruses, in particular microbiome associated phages, viral ageing may promote microbiome dysbiosis with negative consequences for the health (and the ageing) of their own hosts. Although this is absolutely speculative, one could therefore imagine a form of within‐host selection against viral ageing, which could counterselect epigenetic ageing in mutualistic viruses, and/or in viruses with long residency periods within their hosts.

Accordingly, uncovering epigenetic ageing in some viruses might also have potential to unveil unknown efficient viral epigenetic repair systems in others. Because of their sheer numbers and the speed of their replication cycles, viruses evolve particularly rapidly, in contrast to cellular lineages. Consequently, viruses can evolve original molecular systems where other lineages struggle or stall in the face of biological challenges and may have bypassed limits that (multi)cellular life forms cannot surmount. In particular, some viruses that carry a type of epigenetic information similar to our own, yet which do not seem to age, may hold precious molecular keys to our future: original, unexpected viral programs capable of countering the loss of epigenetic signals. Backing up this claim, it is generally accepted that the evolution of viruses has enabled them to develop a certain resistance to epigenetic gene silencing.^[^ ^51^ ^]^ As early as 2008, some researchers were speculating about the existence of viral “demethylation” proteins encoded in adenovirus DNA, or the existence of a viral replication machinery enabling methylation to be avoided.^[^ ^49^ ^]^ More trivially, viruses whose DNA is demethylated after or during viral DNA replication, and that remain in this stage, may implement a form of rejuvenation of their epigenetic age, restoring their original full expression potential.^[^ ^49^ ^]^ An alternative evolutionary solution to the problem of epigenetic Achilles' heels would be for viruses to have developed more reliable, alternative forms of epigenetic regulation, based on chromatin structure for example rather than DNA methylations as is the case for SV40 and other polyomaviruses that display genomes depleted of CpG islands.^[^ ^48^, ^49^ ^]^

Conversely, and not without irony, if the DNA sites with epigenetic information suspected of contributing to mammalian ageing were partly located on genes of viral origin long inserted within their host genomes or came from other “evolving but not living” molecular travelers such as transposons, part of the extant mammalian epigenetic ageing could ultimately be of infectious origin!

Finally, identifying that some viruses could fall prey to age might boost the design of new antiviral therapeutic strategies, that could be developed with the goal to age harmful viruses, including eventually oncogenic viruses. It would provide further justification for a new class of potent, broad‐spectrum antiviral treatments^[^ ^51^ ^]^ that induce a loss of epigenetic signal in our viral parasites, or modify the epitranscriptome of viral RNAs, which can also promote viral replication.^[^ ^51^ ^]^ Studies on the HIV‐1 virus, for example, indicate that, apart from the subject of ageing, playing on viral epigenetic information is a serious therapeutic avenue, since the duration of an infection and that of antiretroviral therapy correlate with the accumulation of methylations in the promoters of this virus.^[^ ^52^ ^]^ Moreover, latent viruses might also be engineered by screening their DNA or RNA for methylations,^[^ ^51^ ^]^ a strategy known as “block and lock”.^[^ ^64^ ^]^

### 1.5 Predictions and ways to test the « ageing viruses hypothesis »

A first prediction of the « ageing virus hypothesis » is that individual viruses that epigenetically age within their host genomes are expected to become replicatively dysfunctional, with a risk to become epigenetically “locked‐in” viruses. This prediction could be tested. For this, one could simultaneously infect clonal host populations with the same virus, and then sequence the viruses' epigenetic information within the DNA of their hosts at regular intervals to track their epigenetic information content. If the viruses integrated into these hosts show increasingly divergent epigenetic profiles with time (e.g., in given regions displaying methylations rather than necessarily at specific DNA sites^[^ ^65^ ^]^), such a divergence would show that the epigenetic signal of these proviruses becomes increasingly heterogeneous as a function of their residence time. If, in addition, an increasing fraction of these viruses with epigenetic variants struggles to extract themselves from their hosts, to resume their lytic cycle, then the variation in their epigenetic information could be correlated with a loss of replicative function, thus with viral replicative ageing. Importantly, host defence mechanisms may underlie both changes in methylation and the resulting functional consequences on viruses. If that were the case, one may wish to exclude such a host effect from the yet‐to‐be‐defined field of viral ageing. It would then be important to distinguish epigenetic ageing in viruses from epigenetic damages on viruses induced by their host's defences. In theory (but that seems difficult in practice), one way to do so would be to study viral ageing in experimentally infected cultured cells from model organisms with very well‐characterized defence mechanisms. Providing such experimental systems are tractable, one could modulate (up or down regulate) the activity of host genes or proteins regulating defence mechanisms with epigenetic targets in these cultured cells and contrast the patterns of epigenetic divergence observed on the genomes of viruses present in such experimentally modified hosts with the patterns of epigenetic divergence observed in viruses from control hosts. Such a comparison could unveil some epigenetic changes that occur independently of the level of expression of the host immune defence and help to establish whether these epigenetic changes are sufficient to prevent the virus to exit to a lytic stage. On the other hand, one may simply consider that host defence mechanisms that result in epigenetic divergence in viruses coupled to an inability of latent viruses to become lytic again are precisely acting as “pro‐ageing” mechanisms that irreversibly manipulate the epigenome of viruses, a situation evoking that observed when a virus like HIV‐1 provokes accelerated host ageing and induces host immunosenescence.^[^ ^66^ ^]^

Alternatively, a more general form of viral ageing could be observed if after a few generations of their hosts, the DNA sequence of epigenetically impaired, hence locked‐in proviruses had accumulated genetic mutations that further prevented the virus from functioning properly, for example, if some of their genes, interrupted by stop codons, became pseudogenes. This phenomenon could be explained by random events and the passage of time; in that case, because it negatively and irreversibly affects the functioning of the virus, it would still deserve to be described as a form of biological ageing caused by a structural weakness: the frailty of the viral epigenome over time. Naming viral ageing this kind of phenomenon could indeed encourage scientists to look for anti‐ageing processes that may evolve in viruses to counteract such an epigenetic signal loss and enhance the search for new anti‐ageing mechanisms.

A second prediction of the « ageing virus hypothesis » is that a certain proportion of sub‐performant viruses, as a result of epigenetic changes but not as a result of genetic changes, are present in nature. This prediction could, for instance, be tested by sequencing the (entire) epigenetic information of double‐stranded environmental DNA viruses—the metavirome epigenome‐, as it is technically possible to obtain DNA methylation profiles of microbial communities living in the ocean.^[^ ^67^ ^]^ The test would be to quantify the proportion of hemi‐methylated, fully methylated or unmethylated CpG islands at a given viral DNA site or in a DNA region of clonal viruses.^[^ ^49^ ^]^ Indeed, 5‐methyl‐Cytosine, the methylated form of the DNA base C, is mainly found in symmetrical dyads of the mCG/GmC type, which can become hemi‐methylated mCG/GC sites, for example during DNA replication, before being restored to their initial state by highly specific DNA methyltransferase enzymes.^[^ ^68^ ^]^ Within a viral population, we expect some epigenetic diversity,^[^ ^69^ ^]^ because distinct methylation profiles can be of no functional importance, can be deleterious, or can have advantages (only) in specific conditions. Importantly, part of this epigenetic diversity may be related to viral ageing, and introduce a certain proportion of sub‐performant viruses, as a result of epigenetic changes but not as a result of genetic changes in an environment or within an infected cell or host. In particular, the proportion of hemi‐methylated sites, potentially carrying erroneous epigenetic information, could provide an approximation of the proportion of ageing viruses in the environment being analyzed.

Hypothetically, epigenomic profiles could be generated for diverse viral populations from viruses grown in a range of culture conditions (e.g., as a histogram of frequency of epi‐alleles for a given population, based on data generated by bisulfite genomic sequencing analysis as in^[^ ^63^ ^]^ or based on data quantifying chromatin structural heterogeneity as in^[^ ^55^ ^]^). Such epigenomic profiles could be compared between different culture conditions (e.g., to assess whether some distributions are enriched in rare epigenetic variants) and to correlate the proportions of epigenetic variants with the proportions of genetic mutations in the tested populations. It might be observed that epigenetic changes rates can be decoupled from genetic changes rates. One could also correlate the proportions of epigenetic variants with the proportions of defective viruses on the one hand and correlate the proportions of genetic variants with the proportions of defective viruses on the other hand in these viral populations. A stronger correlation between proportions of epigenetic changes and proportions of defective viruses than between proportions of genetic changes and proportions of defective viruses would suggest some yet‐to‐be established causality between altered viral epigenetics and viruses defectivity in need for further experimental validation. On the other hand, if viral populations were sampled from a host microbiome, say a mammal individual of known age, it would also be possible to check whether the proportion of hemi‐methylated viral DNA sites increases with host age or with the duration of infection. Such a correlation would suggest that host ageing is a critical feature of viral ageing. Furthermore, the contribution of aged viruses to more or less severe infections could also be tested, typically during massive viral attacks, for example, bursts of viral infections in blooming protists, by correlating the proportion of viruses with significantly hemi‐methylated DNA sites and the severity of infection.

### 1.6 Unsuspected viral causes of animal epigenetic ageing?

There is no doubt that the epigenetic regulation of a diversity of viruses (e.g., gamma herpesviruses such as EBV; HIV, etc.) and of their hosts can be interconnected.^[^ ^60^, ^70^, ^71^, ^72^, ^73^, ^74^ ^]^ For instance, although this is not ageing per se, the Avian leukosis virus has been documented to induce B‐cell lymphoma in chickens by interfering with its host epigenetics. This retrovirus inserts into the TERT promoter region, inhibits the maintenance methylation of that region, which would induce the expression of TERT.^[^ ^75^ ^]^ More to the point, some viruses, such as EBV, evolved specialized weapons able to modulate the epigenetic processes that promote viral genome propagation and host‐cell survival.^[^ ^73^ ^]^ Such viruses can therefore interfere at an epigenetic level on the persistence, hence on the chronological ageing of their host cells.

But more generally, one might wonder whether, in some cases mammalian epigenetic ageing could have an infectious origin. Ancient infections, that is, infections that occurred before the birth of a focal ageing organism, for example, an infection that resulted in the long‐term insertion of viral sequences within the genomes of a host lineage, may theoretically be involved in the subsequent developments of animal epigenetic ageing. In our species, prime suspects feature human endogenous retroviruses (HERV), known to influence our gene transcription,^[^ ^76^ ^]^ possibly by epigenetic interferences.^[^ ^77^ ^]^ Whether ancient viral integrations may have impacted animal epigenetic ageing may be tested in (at least) two ways. First, individual phylogenetic trees of the host components of the present‐day epigenetic signal repair and maintenance machinery might show evidence of some lateral transfer from viruses. In that case, the host sequences coding for that machinery would be more closely related to viral sequences than to their usual eukaryotic homologs. Such a pattern would suggest that critical components of “irremediable” animal epigenetic ageing according to ETA^[^ ^30^ ^]^ would be of viral origins, because some genes identified by Duffield et al.^[^ ^30^ ^]^ as being associated with ageing through epigenetic deregulation would have been laterally acquired. Likewise, individual phylogenetic trees of the host components of an epigenetic‐based developmental program, whose break has been hypothesized to contribute to animal ageing either by antagonistic pleiotropy^[^ ^25^ ^]^ or via cellular dedifferentiation^[^ ^31^ ^]^ may show evidence of lateral gene transfer from viruses. However, such phylogenetic tests of a hypothetical hidden viral contribution to animal ageing would still require experimental validations to determine whether the supposedly laterally acquired genes affect ageing when knocked‐out or edited.

Likewise, more contemporary events of viral infection leading to animal epigenetic ageing could be experimentally tested. In a nutshell, it is known that viral infection changes the epigenetic profile of the infected host, as the integration of foreign DNA can result in changes of DNA methylation and of transcription patterns in mammalian cells.^[^ ^78^ ^]^ For instance, Adenovirus infections deregulate the expression of cellular genes, many of which are repressed in less than 24 h, due to changes in epigenetic regulation, following the insertion of foreign viral DNA into the host DNA molecule. In particular, the methylation state of Adenoviruses and of the DNA adjacent to their insertion site changes. This new epigenetic state in the host is maintained even after the infectious virus has disappeared.^[^ ^48^ ^]^ Likewise, EBV infection can induce an epigenetic mutator phenotype in its host and these epigenetic alterations are retained even after the loss of the virus.^[^ ^79^ ^]^ More precisely, viral proteins such as EBV's Epstein‐Barr virus nuclear antigen (EBNA) or KSHV's LANA (latency‐associated nuclear antigen) proteins are known to interact with the chromatin structures of their hosts, potentially contributing to the development of various types of cancer,^[^ ^80^ ^]^ hinting that some viruses may negatively affect their host fitness by their epigenetic destabilizing effects.^[^ ^81^, ^82^ ^]^ A faster ticking of these clocks could indicate that the epigenome of the infected host has changed in ways similar to that usually seen at older calendar ages in non‐infected hosts and suggest an infectious origin of their ageing.

## 2 CONCLUSION

Ageing is a natural outcome of evolution, either directly (according to adaptive theories of ageing) or indirectly (according to mainstream non‐adaptive theories of ageing). To maintain their identity and functions, cells and organisms rely upon evolved biological processes able to preserve their information content for a certain time in the face of entropic diffusion. Beyond cellular organisms, viruses, that are also evolved biological entities, are likely subjected to similar constraints.

However, traditional theories on ageing largely ignore viruses. They are not considered likely to age, perhaps because biologists debate whether viruses are living or non‐living, and it may be hard to imagine that something that is not alive ages. On the contrary, the recent development of epigenetic theories of ageing makes it possible to imagine that even viruses age, because after all, nobody disputes that viruses evolve, that is, that many aspects of their optimal functioning can be explained by natural selection, and that some viruses use epigenetic information systems for the optimal completion of their replication cycle and persistence. If it were verified that some viruses are epigenetically ageing in their own right, this would demonstrate that ageing is more universal than currently acknowledged and anticipated in the evolving biological world and affects more than cells or organisms. Viruses would then deserve a greater, direct, consideration by theories of ageing, beyond their current proposed contribution to the ageing of other entities, namely their hosts. So far, viruses have been at best considered either as parasites that may affect the evolution of host longevity programs due to the selective pressures that they impose upon their hosts lineages and due to the threat that viruses raise for the sustainability of their host population dynamics.^[^ ^83^ ^]^ Or, more recently, some viruses have been predicted to act as host age‐distorters^[^ ^44^, ^45^ ^]^ by altering the normal course of ageing of their infected hosts. The ageing virus hypothesis, by contrast, makes viruses ageing agents in their own right.

Moreover, if this hypothesis is correct, epigenetic loss of information would have been amongst the first causes of ageing to appear in the natural history of ageing, possibly along with the loss of proteostasis, currently assumed to be the oldest form of ageing.^[^ ^84^ ^]^ By contrast, other possible mechanisms of ageing (e.g., cellular senescence, mitochondrial dysfunction, etc.) would have evolved later. If indeed, epigenetic ageing was amongst the earliest evolved causes of ageing, one could further speculate that by setting a limit to the “healthspan” of both viruses and their hosts, epigenetic ageing may have contributed to the subsequent evolution of both anti‐ageing and ageing mechanisms (which may or may not comprise an epigenetic component) in epigenetically endowed lineages.

Some common objections may speak against the plausibility of the hypothesis. First, not all viruses would extensively use epigenetics in their normal functioning. Second, would we be able to uncover viruses that age, whereas such viruses may seem at risk to disappear? Third, is there specific evidence that some viruses would cause organisms to age epigenetically, or that their hosts may accelerate the ageing of these viruses? In my opinion, the answers to these three objections bring support to the ageing virus hypothesis. First, a broad diversity of viruses relies on epigenetic information. So, at least some viruses would be concerned by this hypothesis. Second, ageing in a viral population or lineage need not to lead this viral population or lineage to extinction. Consequently, even ageing viruses could in principle be observed. In fact, many lineages composed of biologically ageing organisms, whose populations show positive senescence, just like humans, have clearly not gotten extinct. Moreover, neither adaptive nor non‐adaptive theories on the evolution of ageing predict that ageing will lead a species to extinction. The former theories consider ageing as an adaptation that would benefit its host lineage, whereas non‐adaptive theories see ageing as a collateral damage (often of a successful individual reproduction). There is therefore no reason to expect that ageing viruses would be eliminated from the planet. Third, several findings indicate that at least some viruses likely cause their hosts to age epigenetically. This has been notably proposed for HIV‐1.^[^ ^66^, ^85^ ^]^ Of course, however, because the idea that viruses could age is novel, the often described reports of viral epigenetic signal modifications induced by their hosts have not been previously associated with the possibility that some hosts may accelerate viral ageing.

**FIGURE 1:**
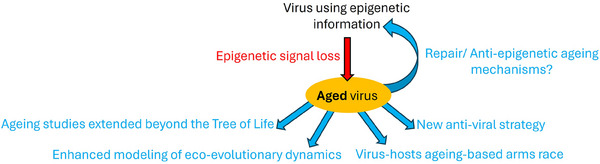
The “ageing virus hypothesis” and its implications. If according to the ageing virus hypothesis some viruses age, a new phenotype: “viral ageing” induced by epigenetic signal loss (orange ellipse) could be investigated with multiple scientific potential pay‐offs (in blue).

Therefore, uncovering ageing viruses might profoundly change our views on the amount of ageing biological entities on the planet (Figure 1). In fact, the discovery of epigenetically ageing viruses would improve no less than: virology, by identifying a new viral phenotype ; gerontology, by extending the scope of ageing theories to the most abundant and biologically central entities on Earth, and possibly by unveiling new viral mechanisms able to modulate epigenetic ageing and rejuvenation; evolutionary biology, by improving current evolutionary theories of ageing through an explicit characterization of the contribution of viruses to the evolution of ageing; molecular ecology and genomics, by taking into account new parameters to explain microbial dynamics and pandemics in nature and in host genomes; and perhaps even medicine, if, in the future, some of the viruses that cause us to age can be defeated by some new treatments that would make them age first!

The possibility that some viruses age epigenetically raises numerous novel research questions. The development of models to describe epigenetic signal loss in virus infecting a host is one of them. This is especially complicated as it requires to distinguish self‐referential epigenetic dynamics of viruses and hosts from tight integration of virus life cycles with host physiology, and to disentangle functional changes in epigenetic signal accompanying the progression across the viral cycle (e.g., between latent and lytic phases) from changes leading to viral epigenetic ageing. It would also likely benefit from considerations on the time frames at which viral epigenetic ageing is expected to be observed, when it is driven by viral processes rather than by the integration into the epigenetic signal loss process of the host, which themselves may vary with host age.

## CONFLICT OF INTEREST STATEMENT

The authors have no conflicts to disclose.

## Data Availability

Not applicable.
